# Educational quality of YouTube content on healthcare waste management relevant to dentistry: a cross-sectional study

**DOI:** 10.1186/s12903-026-10137-4

**Published:** 2026-10-12

**Authors:** Filipe Lima Goetz Fagundes, Daniel Costa Magalhães da Cunha, João Victor Frazão Câmara, Aléxia Caroline Leandro da Conceição, Victor de Macedo Varela, Gisele Damiana da Silveira Pereira

**Affiliations:** 1 College of Dentistry, Federal University of Rio de Janeiro, Rio de Janeiro, RJ Brazil; 2 Saarland University, Clinic of Operative Dentistry, Periodontology and Preventive Dentistry, Building 73, Homburg Saar, 66421 Germany

**Keywords:** Health education, Healthcare waste management, Dentistry, YouTube

## Abstract

**Background:**

YouTube has become a major source of educational content in healthcare, including topics related to dentistry and public health. However, the accuracy and educational reliability of videos addressing healthcare waste management relevant to dental practice remain uncertain. Therefore, the aim of this cross-sectional study was to evaluate the educational quality, credibility, and reliability of YouTube videos addressing healthcare waste management with relevance to dental practice, using the Global Quality Scale (GQS), Journal of the American Medical Association (JAMA) benchmark criteria, and the modified DISCERN (mDISCERN) instrument. Additionally, the influence of video source type and the correlation among the evaluation instruments were investigated.

**Methods:**

A cross-sectional analysis was conducted in June 2025 using Portuguese search terms related to healthcare waste management, including “Resíduos de Serviço de Saúde,” “Resíduos Hospitalares,” and “Resíduos Odontológicos,” to identify videos relevant to dental practice. A total of 180 YouTube videos were initially screened. After duplicate removal and application of inclusion criteria, 47 videos were included for final analysis. Two independent and calibrated reviewers assessed all videos using the Global Quality Scale (GQS), Journal of the American Medical Association (JAMA) benchmark criteria and modified DISCERN (mDISCERN) instrument. To account for the Brazilian regulatory context, particularly Resolution RDC No. 222/2018, the GQS, JAMA benchmark criteria, and mDISCERN were applied as contextually adapted applications of the original instruments, using study-specific operational definitions to guide the interpretation of selected criteria. These contextual adaptations preserved the general structure and scoring ranges of the instruments but may have altered the interpretation of some underlying constructs. Because these adaptations have not undergone independent psychometric validation, they should not be considered validated modifications of the original instruments, and the findings should be interpreted within the specific methodological context of the present study. Inter-rater reliability was evaluated using the intraclass correlation coefficient (ICC) and Cronbach’s alpha. Descriptive analyses were performed, followed by ANOVA and Kruskal–Wallis tests to compare scores according to source type (academic institutions, organizations, healthcare professionals, and other users). Spearman’s correlation test assessed associations among evaluation instruments.

**Results:**

Excellent inter-rater agreement was observed (ICC = 0.946; 95% CI: 0.913–0.967; Cronbach’s alpha = 0.913). Mean scores obtained using the contextually adapted applications indicated high quality of the assessed educational content according to the GQS (4.19 ± 0.78), moderate credibility/transparency (JAMA: 2.43 ± 1.06), and moderate reliability (mDISCERN: 2.50 ± 0.90). No statistically significant differences were identified among source types for any scale (*p* > 0.05). A significant positive correlation was found between JAMA and mDISCERN scores (ρ = 0.502; *p* < 0.001), indicating an association between these scores within the contextually adapted measurement framework. No significant correlations were observed between GQS and the other instruments.

**Conclusions:**

The 47 YouTube videos analyzed in the present study demonstrated satisfactory quality of the assessed educational content but only moderate credibility and reliability, based on the contextually adapted applications of the GQS, JAMA benchmark criteria, and mDISCERN. Because the contextual operational definitions used for these instruments have not undergone independent psychometric validation, the results should be interpreted within the methodological framework adopted in this study. No statistically significant differences in scores were detected among the represented source categories. However, a positive association was observed between the scores obtained using the contextually adapted JAMA and mDISCERN applications, whereas GQS scores were not significantly associated with either instrument. These findings underscore the importance of critically evaluating online health-related content using assessment approaches that address different dimensions of quality. The results describe the characteristics of the videos retrieved under the specified search strategy during the June 2025 data collection period and should not be generalized to all YouTube content or interpreted as evidence of educational effectiveness, learning outcomes, professional competence, or changes in healthcare waste management practices. Furthermore, whether the characteristics identified in these videos translate into measurable educational benefits or contribute to learning and professional training remains to be determined through direct evaluation in future educational studies.

**Supplementary Information:**

The online version contains supplementary material available at https://doi.org/10.1186/s12903-026-10137-4.

## Introduction

Healthcare waste (HCW) comprises discarded materials generated from activities related to human healthcare, including clinics, hospitals, laboratories, mortuaries, funeral services, research centers, and health education institutions [1, 2]. Because these materials frequently contain hazardous biological, chemical, and sharps components, they pose substantial risks to healthcare workers, the general population, and the environment when improperly managed. Consequently, the World Health Organization classifies healthcare waste as one of the most significant categories of hazardous waste worldwide [3]. Effective management of HCW therefore requires strict compliance with protocols for segregation, packaging, transportation, treatment, and final disposal to minimize environmental contamination and prevent disease transmission [1, 2].

In Dentistry, healthcare waste generation is continuous and highly heterogeneous, encompassing common solid waste, infectious materials, and sharps, which demand rigorous biosafety practices [4]. Dental services routinely generate potentially hazardous residues, including heavy metals from radiographic solutions and dental amalgam, toxic chemical disinfectants and sterilizing agents, as well as contaminated cotton, plastics, latex, glass, needles, and blades exposed to biological fluids [4]. Inadequate management of these materials may result in occupational accidents and environmental contamination, affecting not only oral healthcare professionals but also waste collectors, cleaning personnel, and the wider community. Injuries involving contaminated sharps may facilitate the transmission of serious infectious diseases, including HIV and viral hepatitis, highlighting the critical importance of safe and responsible waste management practices in dental education and clinical care [5].

In Brazil, the Agência Nacional de Vigilância Sanitária and the Conselho Nacional do Meio Ambiente require all healthcare institutions to implement a Healthcare Waste Management Plan (Plano de Gerenciamento de Resíduos de Serviços de Saúde – PGRSS). These regulations establish a set of technical and operational procedures aimed at ensuring the safe and efficient management of healthcare waste, including segregation, packaging, identification, temporary storage, preliminary treatment, occupational safety measures, transportation, and environmentally appropriate final disposal [1, 4, 6]. Effective implementation of these procedures is essential to reduce environmental contamination and protect public health.

Given the complexity and risks associated with dental healthcare waste, oral health professionals must be adequately trained and aware of their responsibilities regarding biosafety and environmental sustainability. Therefore, undergraduate dental education should provide students with robust knowledge of infection control protocols, healthcare regulations, and ethical responsibilities related to waste management practices [1, 4, 6]. Nevertheless, despite the existence of well-established guidelines, partial or complete noncompliance with recommended practices remains common in clinical settings. Factors such as insufficient technical knowledge, negligent attitudes, and inadequate infrastructure contribute to unsafe working conditions and increase the risk of contamination and occupational exposure among healthcare workers, waste handlers, and the broader community.

Simultaneously, the rapid expansion of internet access and digital media platforms has transformed how healthcare information is disseminated and consumed. Students, healthcare professionals, and the general public increasingly rely on online video-sharing platforms as educational resources for topics related to dentistry, biosafety, and risk prevention [7–9]. Among these platforms, YouTube has become one of the most widely used free educational tools worldwide due to its accessibility, broad audience reach, and multimedia capabilities [10–13]. However, the absence of rigorous peer-review mechanisms and formal content verification raises concerns regarding the scientific accuracy, credibility, and reliability of the information shared online. Because any user may publish content without expert validation, misleading, outdated, or scientifically unsupported information can be easily disseminated, potentially compromising professional training and evidence-based practice [9, 14, 15].

Despite the growing body of literature evaluating YouTube as a source of health-related educational information, no previous study has specifically investigated the educational quality, credibility, and reliability of YouTube videos addressing healthcare waste management relevant to dentistry. Therefore, the aim of this cross-sectional study was to evaluate the educational quality, credibility, and reliability of YouTube videos on healthcare waste management relevant to dental education and clinical practice.

## Materials and methods

### Study design

This quantitative, observational, cross-sectional study was based on the content analysis of publicly accessible videos published on the Brazilian version of YouTube. This study was conducted and reported according to the Strengthening the Reporting of Observational Studies in Epidemiology (STROBE) guidelines for cross-sectional studies. The unit of analysis was the individual YouTube video, and the study evaluated publicly available educational content related to healthcare waste management relevant to dentistry.

The study was conducted on the Brazilian version of YouTube, and video searches were performed in June 2025, using the Portuguese-language keywords “Healthcare Waste”, “Hospital Waste”, and “Dental Waste” to identify YouTube videos addressing healthcare waste management with relevance to dental education and clinical practice. These search terms were intentionally selected to capture both general healthcare waste management content and dentistry-specific educational material, as dental waste management is regulated within the broader framework of healthcare waste management in Brazil [11, 13, 14, 16, 17].

### Identification of search keywords

To identify the most frequently used search terms related to the study topic, the term “Health Waste” was initially explored using Google Trends (https://trends.google.com/trends) [9, 18]. This platform enables analysis of the relative frequency of specific search queries in relation to the total search volume across different regions and time periods, allowing the identification of the most searched terms within a particular topic area (https://trends.google.com/trends). After comparing related expressions, the term “Healthcare Waste” was identified as the most frequently searched keyword during the evaluated period, followed by “Hospital Waste” and “Dental Waste”. These terms were subsequently selected to guide the video search strategy [9, 17, 18].

### Search strategy

An examiner (FLGF) conducted the video search on YouTube using the Google Chrome browser in incognito mode, with browser cache and cookies cleared and all Google accounts logged out, to minimize potential interference from personalized recommendation algorithms. In addition, a newly created YouTube account was used to further reduce the influence of previous user interactions or viewing preferences on search results. To avoid interruptions caused by advertisements during data collection, an ad-blocking application (AdBlock – AdBlock Inc., Houston, USA) was activated throughout the search process [13–15, 18–21]. No search filters were applied to avoid restricting the retrieval process. Consequently, video results were automatically ranked according to the relevance algorithm active on YouTube at the time of data collection [9, 13, 15, 20].

Several strategies were adopted to minimize potential selection and information bias during video retrieval and screening. These included the use of an incognito browser mode, clearing browser cache and cookies, creating a new YouTube account, and conducting the initial screening while the evaluator was blinded to video engagement metrics, such as likes, dislikes, and comments. These procedures aimed to reduce the influence of personalized recommendations, previous user interactions, and popularity indicators on the selection and evaluation of videos.

The search strategy included the following Portuguese-language terms: “Healthcare Waste”, “Hospital Waste”, and “Dental Waste”. These terms were selected because of their strong relevance to the study topic, encompassing both general and dentistry-specific aspects of healthcare waste management, as well as their high search frequency on YouTube [15]. The search yielded 788 videos for “Healthcare Waste,” 461 for “Hospital Waste,” and 137 for “Dental Waste.” From the videos retrieved for each search term, the first 60 videos displayed according to YouTube’s default relevance-based search ordering were selected, resulting in an initial sample of 180 videos. The videos were identified and selected according to YouTube’s default relevance-based search ordering, and no additional criteria based on engagement metrics, such as number of views, likes, or comments, were applied during the selection process. This approach was based on evidence indicating that most YouTube users typically access only the first 60 videos displayed in search results, with many restricting their viewing to the first 30 results [14]. Similarly, internet search behavior studies suggest that users rarely navigate beyond the first five pages of search results, corresponding to approximately 30 links per page [21]. Considering these browsing patterns, the first 180 videos retrieved according to YouTube’s default relevance-based search ordering (60 videos for each search term) were included for subsequent screening, representing the content most prominently displayed by the platform during the data collection period [15–17, 22–24].

### Sample size calculation

The minimum sample size was estimated based on parameters derived from previous YouTube-based studies. Alsulaiman et al. (2024) examined the quality of oral hygiene education videos on YouTube and reported mean quality scores of 3.60 (SD = 0.50) for high-quality content and 2.99 (SD = 0.59) for low-quality content. Using these values in G*Power (version 3.1.9.2; Heinrich Heine University, Düsseldorf, Germany), with a Type I error rate of α = 0.05 and a statistical power of 80%, a minimum of 10 videos per group was estimated, corresponding to a Cohen’s d effect size of 1.4. Similarly, Ozdede and Peker (2020) estimated a total minimum sample of 116 videos for a comparable design, based on a medium effect size (Cohen’s d = 0.40), 85% power, and α = 0.05. The final sample was determined through systematic application of the eligibility criteria to the first 180 videos retrieved according to YouTube’s default relevance-based search ordering identified across three search terms (“Resíduos de Serviço de Saúde”, “Resíduos Hospitalares”, and “Resíduos Odontológicos”- 60 videos for each search term). After screening, 47 videos met the inclusion criteria and were retained for analysis.

### Video storage

180 selected videos were electronically saved using the playlist feature of YouTube. The videos were distributed into three separate playlists, each containing 60 videos, and organized in descending order of relevance to facilitate future systematic analyses. The Uniform Resource Locators (URLs) of the selected videos were copied and electronically stored in a spreadsheet using Microsoft Excel (Microsoft Corporation, Redmond, USA), also arranged in descending order of relevance to enable systematic future analyses. The URLs were preserved to ensure data traceability.

### Video screening

Video screening was conducted by a single evaluator (FLGF), who underwent prior training to ensure consistency in the application of video eligibility criteria. Initially, all stored videos were screened to identify and remove duplicates. Duplicate videos were defined as videos originating from the same source but uploaded at different time points; in such cases, only the earliest version was retained. As a result of this process, 22 videos (*n* = 22) were excluded, leaving 158 videos (*n* = 158) for further analysis [9, 20, 23].

In the subsequent stage, the same evaluator performed an additional screening process in which the predefined inclusion and exclusion criteria were applied, ensuring methodological consistency and the relevance of the final sample analyzed [9, 20, 23]. To minimize potential bias during the initial screening process, the evaluator was blinded to the number of likes, dislikes, and comments associated with the videos, thereby preserving the objectivity of the analysis [16, 18].

### Eligibility criteria

Following duplicate removal, the inclusion and exclusion criteria were applied to the remaining 158 videos [13, 15, 24]. Videos were included if they were presented in Brazilian Portuguese, had satisfactory audio and video quality, including a minimum resolution of 240p and preferably 720p HD quality [15, 17], and contained informative content directly related to the study topic. Videos presented by healthcare professionals, academics, patients, or family members were considered eligible. Additionally, only videos published within the last 10 years were included in the analysis.

Videos were excluded if they were presented in languages other than Brazilian Portuguese [16], lacked specific or relevant content related to the study topic [12, 14, 18], had no title, consisted exclusively of audio or video content, or demonstrated unacceptable audiovisual quality [15]. As were livestreams, short-form videos, animated GIFs, image compilations, book or article reviews, promotional product websites, membership-based websites, discussion groups, video feeds, and commercial or advertising materials, including assistive technology advertisements, news reports, advertisements, and music videos [13, 22]. Videos longer than 30 min were also excluded [18]. Longer videos, particularly extended lectures, webinars, or complete training sessions may differ substantially from the shorter educational content commonly accessed by YouTube users and may include multiple topics beyond the specific scope of healthcare waste management relevant to dentistry [18].

**Fig. 1:**
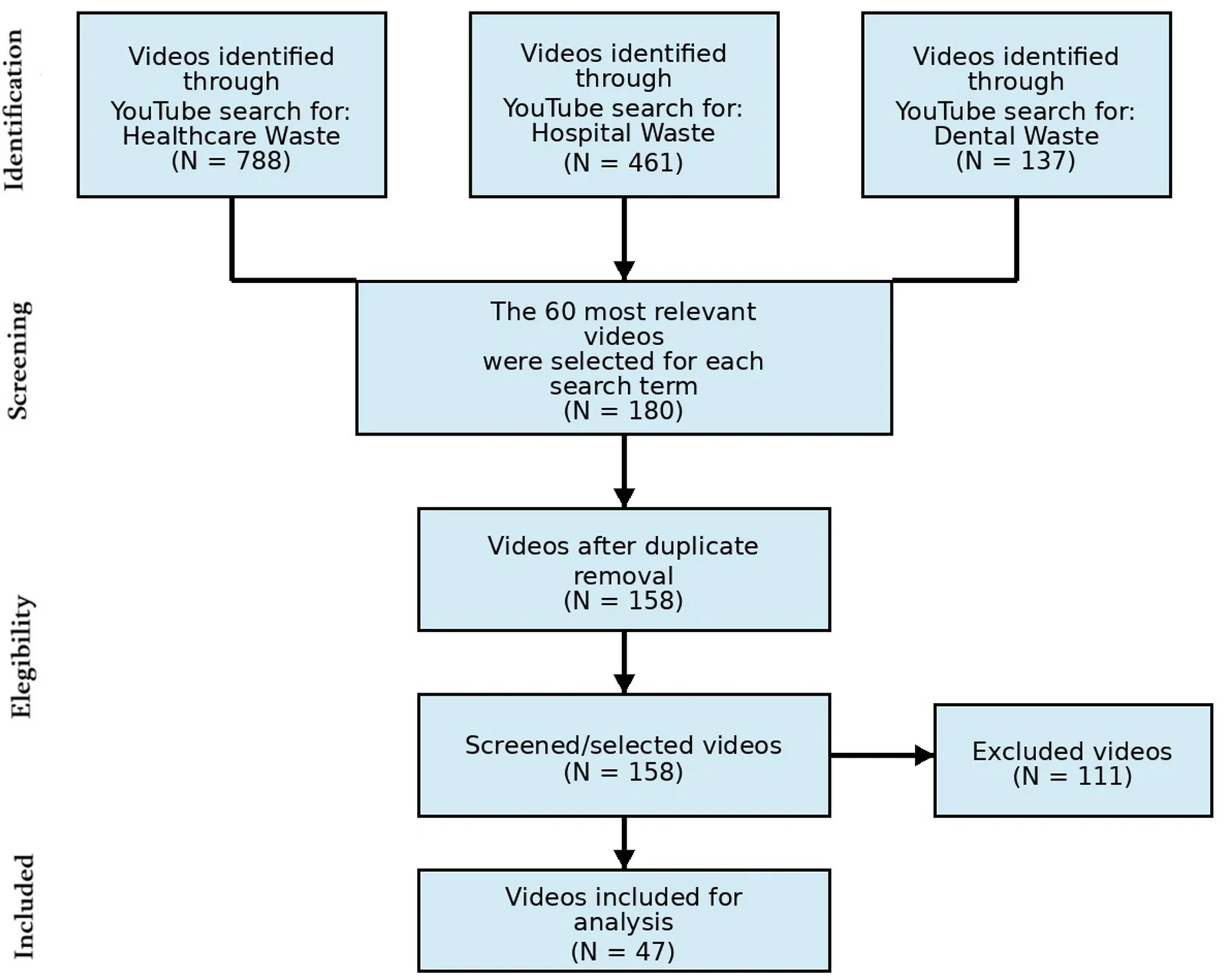
Flow diagram of the sample selection process, detailing the stages of search and identification, screening, and eligibility of the videos used in the study

After the application of all eligibility criteria, a final sample of 47 videos (*n* = 47) was retained for subsequent analysis (Fig. 1).

### Data collection and video variables

All videos remaining after the initial screening process were fully viewed and independently analyzed by the same evaluators. For each video, the relevant data were systematically collected and recorded in a Microsoft Excel spreadsheet. The variables extracted included demographic characteristics of the videos, specifically the title, URL, and upload source [11, 16, 20, 23, 24].

In addition, videos were classified according to their source of production, based on the authorship or institutional affiliation responsible for the uploaded content [11, 12, 14, 16, 20–24]. The videos were categorized into six groups: (1) academic sources, including content affiliated with research groups, universities, dental schools, or other health-related academic institutions; (2) professional healthcare organizations, such as hospitals, clinics, professional councils, dental associations, and the Ministry of Health; (3) healthcare professionals, including general dentists, dental specialists, and other healthcare providers; (4) dental information sources, comprising websites dedicated to dental education and information dissemination; (5) non-governmental organizations (NGOs); and (6) other individual users, including members of the general public, students, or professionals not associated with the healthcare field. All predefined source categories were considered during video classification. However, only categories represented in the final sample were included in the comparative statistical analyses. Categories with no eligible videos were retained during the classification process but were not analyzed due to the absence of observations.

### Video evaluation

The quality, credibility, and reliability of the YouTube™ videos were assessed using specific evaluation instruments, including the Global Quality Scale (GQS), the Journal of the American Medical Association (JAMA) benchmark criteria, and the modified DISCERN (mDISCERN) instrument, to avoid overlap between assessment domains and ensure methodological clarity.

To ensure greater standardization and consistency in the evaluation process, two evaluators (FLGF and DCMC) underwent a structured training and calibration procedure. Initially, both evaluators watched instructional videos explaining the assessment scales used in the study [13]. Subsequently, calibration exercises were conducted, including independent evaluations followed by consensus discussions, as well as the joint assessment of five videos related to the study topic. Disagreements were discussed between the two evaluators until consensus was reached. No third evaluator was consulted, as consensus was achieved through discussion between the two reviewers [13].

Because no validated assessment instrument is specifically available for evaluating healthcare waste management educational content relevant to dentistry, the GQS, JAMA benchmark criteria, and mDISCERN were used as contextually adapted applications of the original instruments. Study-specific operational definitions were established a priori during the calibration process to guide evaluator interpretation and improve consistency within the specific regulatory and educational context of healthcare waste management in Brazil. Although the original scoring ranges and general structures of the instruments were retained, selected criteria were operationalized according to predefined contextual rules. Therefore, these applications should not be considered validated modifications of the original instruments, and the resulting scores may represent constructs that differ, to some extent, from those assessed by the original validated versions. Accordingly, direct comparisons with studies using the original instruments according to their unmodified definitions should be made with caution.

#### Assessment of the educational quality of informative videos – Global Quality Scale (GQS)

The educational quality of the 47 selected videos was assessed using the Global Quality Scale (GQS), an instrument developed by Bernard et al. (2007) [25]. This tool consists of five evaluation criteria (Supplementary material 1) and enables the classification of videos according to the quality, clarity, coherence, and flow of the information presented, as well as its relevance and applicability to the target audience [10–13, 15, 16, 20].

Each video received an individual score based on a five-point Likert scale, with ratings ranging from 1 to 5 according to the criteria established by the instrument. A score of 5 represented the highest educational quality, indicating full compliance with the evaluated criteria [10–13, 15, 16, 20].

To improve standardization and facilitate evaluator judgment, predefined contextual criteria related to essential healthcare waste management topics, including classification, segregation, and packaging/storage, were used during GQS assessment. These criteria were incorporated as study-specific contextual operational definitions to guide the interpretation of educational relevance and applicability within the dental healthcare waste management context. Although the original five-point GQS scoring range was retained, the use of these contextual criteria may have influenced the interpretation of the GQS construct in the present study. Therefore, the GQS scores should be understood as a contextually adapted application of the original instrument rather than as a fully unmodified application of its validated definition. Accordingly, comparisons with studies applying the original GQS according to its unmodified validated definition should be made with caution.

Based on the original GQS classification, scores of 1–2 were categorized as low quality, a score of 3 as intermediate quality, and scores of 4–5 as good to excellent quality [18, 19, 24]. This classification was retained to describe the distribution of GQS scores and was not considered an investigator-defined cut-off. For the secondary dichotomized sensitivity analysis, scores of 1–3 were grouped as lower quality and scores of 4–5 as higher quality. This 1–3 versus 4–5 grouping was defined a priori by the investigators solely for exploratory purposes and does not represent the original categorical classification or a validated GQS threshold.

#### Assessment of the reliability of informative video sources – JAMA benchmark criteria

The reliability of the video sources was evaluated using the benchmark criteria proposed by the Journal of the American Medical Association (JAMA), originally developed by Silberg et al. in 1997. Considered one of the most simplified quality assessment tools, the JAMA criteria enable the rapid identification of websites and online content, lacking basic standards of transparency and reliability.

The JAMA benchmark criteria (Supplementary material 2) were applied to assess the reliability of the videos using a binary “yes” or “no” scoring system for each evaluation criterion. Each affirmative response (“yes”) was assigned one point, whereas negative responses (“no”) received no score contribution. The final score ranged from 0, indicating the lowest level of reliability, to 4, representing the highest level of reliability and transparency, based on the presence of four fundamental standards of information quality, each weighted equally with one point [12]. For exploratory analyses, JAMA scores were categorized according to the number of fulfilled criteria. Because the original JAMA framework does not establish a validated dichotomous threshold, scores of 1–2 were classified as lower fulfillment and scores of 3–4 as higher fulfillment for the present exploratory sensitivity analysis. This dichotomization was defined a priori by the investigators solely to facilitate comparisons with the other assessment instruments and should not be interpreted as an established categorical classification of the original JAMA framework.

To ensure a more objective and standardized transparency assessment, operational definitions were established during the calibration phase to improve consistency in the interpretation of JAMA criteria within the context of healthcare waste management applicable to dentistry. The original domains and scoring system of the JAMA benchmark criteria were maintained.

The JAMA benchmark criteria were therefore applied as a contextually adapted application of the original framework. The “Authorship” criterion was evaluated according to its original definition because it was considered sufficiently clear and objective. In contrast, the remaining criteria were operationalized using study-specific rules developed a priori to reduce subjectivity and to reflect the Brazilian regulatory context. For the “Currency” criterion, a contextual regulatory rule was adopted whereby a positive score required explicit reference to Resolution RDC No. 222/2018, which was considered the principal regulatory reference for healthcare waste management in Brazil during the study period. The “Attribution” criterion was operationalized as the clear identification or citation of the sources supporting the information presented, whereas the “Disclosure” criterion focused on the identification of the individuals, institutions, or sponsors responsible for the video content.

These operational definitions were developed specifically for the present study and may have changed the interpretation of the corresponding JAMA constructs relative to their original definitions. Thus, although the four-item structure and 0–4 scoring range of the JAMA benchmark criteria were retained, the resulting scores represent a contextually adapted application rather than an unmodified application of the original JAMA criteria. These operational definitions have not undergone independent psychometric validation and should not be interpreted as validated modifications of the original instrument. Comparisons with studies applying the original JAMA benchmark criteria according to their unmodified definitions should therefore be made with caution.

#### Assessment of the reliability of informative video content – DISCERN scale

For the present study, the modified DISCERN instrument (mDISCERN), adapted by Pons-Fuster et al. (2020), was used [26]. This shortened version consists of five questions designed to assess the reliability of online health information [11–13, 15, 16]. The mDISCERN criteria were evaluated using a binary “yes” or “no” classification system for each of the five questions. Each affirmative response received one point, whereas negative responses received no score contribution, resulting in a final score ranging from 0 to 5, with higher scores indicating greater reliability [11]. For the present study, no validated categorical threshold was identified for the mDISCERN application used. Therefore, for the exploratory sensitivity analysis, scores of 1–3 were grouped as lower reliability and scores of 4–5 as higher reliability. This 1–3 versus 4–5 grouping was defined a priori by the investigators solely for exploratory purposes and does not represent an original or validated categorical threshold of the mDISCERN instrument.

To provide a more objective and reproducible assessment process, the five mDISCERN questions (Supplementary material 3) were accompanied by predefined interpretation guidelines established during calibration to improve consistency among evaluators.

For the present study, the mDISCERN was therefore applied as a contextually adapted application of the original instrument. The original five-item structure and binary scoring system were retained; however, predefined study-specific interpretation guidelines were developed during calibration for each criterion to improve consistency within the Brazilian healthcare waste management context. For the first criterion, a positive score was assigned when the video clearly stated its objective. For the second criterion, a positive score required explicit identification or citation of information sources. The third criterion, concerning balance and impartiality, was operationalized according to predefined contextual guidance requiring consistency between the information presented and applicable ANVISA healthcare waste management regulations. The fourth criterion required the provision of additional resources or references for further learning. The fifth criterion, concerning areas of uncertainty, was operationalized according to the study-specific framework by identifying whether the video acknowledged limitations, uncertainties, or complementary aspects beyond the baseline recommendations established by Resolution RDC No. 222/2018.

Each criterion fulfilled according to these predefined rules contributed one point to the final mDISCERN score, resulting in a total score ranging from 0 to 5. These interpretation guidelines were developed exclusively for the present study and have not undergone independent psychometric validation. Accordingly, they should not be considered validated modifications of the original mDISCERN instrument, and the resulting scores should be interpreted as a contextually adapted application whose construct validity and comparability with studies using the original mDISCERN definitions may be limited.

### Statistical analysis

Statistical analyses were performed using IBM SPSS Statistics software, version 29.0, and Jamovi software, version 2.6.44. SPSS Statistics was used for Cronbach’s alpha calculations, whereas Jamovi was used for the Intraclass Correlation Coefficient (ICC), one-way analysis of variance (ANOVA), Kruskal–Wallis test, and Spearman’s correlation analyses.

Data analysis was organized in four stages: assessment of inter-rater reliability, descriptive analysis of the sample, comparison of evaluation scores among author/source categories, and correlation analysis among the assessment instruments.

Inter-rater reliability was assessed using the ICC, calculated for the two independent raters across the 47 videos, and Cronbach’s alpha was calculated as a complementary measure of internal consistency between raters. The interpretation of ICC values followed the classification proposed by Cicchetti (1994), in which values < 0.40 indicate poor agreement, values between 0.40 and 0.59 indicate fair agreement, values between 0.60 and 0.74 indicate good agreement, and values ≥ 0.75 indicate excellent agreement [13]. The mean of the two raters’ scores was used in all subsequent analyses.

Descriptive statistics are presented as means, standard deviations, frequencies, and percentages. Differences in GQS, JAMA, and mDISCERN scores among author/source categories were evaluated using one-way ANOVA or Kruskal–Wallis tests after assessment of normality (Shapiro–Wilk) and homogeneity of variance (Levene), as appropriate. Test statistics, degrees of freedom, effect sizes (η² and ω² for ANOVA; ε² for Kruskal–Wallis), and 95% confidence intervals are reported for group means and for Spearman’s correlation coefficients.

Associations among the evaluation instruments were assessed using Spearman’s correlation coefficient. Continuous scores were used for the primary analyses, whereas dichotomized versions of the scales were examined only as secondary exploratory (sensitivity) analyses to facilitate interpretation of potential relationships. For each instrument, the basis of the categorical thresholds was specified according to the corresponding literature or, where no established categorical threshold was identified, defined a priori for the present exploratory analysis.

For the GQS, the original classification described scores of 1–2 as low quality, 3 as intermediate quality, and 4–5 as good to excellent quality, consistent with classifications reported in previous studies [18, 19, 24]. These categories were used to describe the score distribution and were not treated as investigator-defined thresholds. For the secondary sensitivity analysis, scores of 1–3 were grouped into a lower category and scores of 4–5 into a higher category. This 1–3 versus 4–5 dichotomization was defined a priori by the investigators solely for exploratory purposes and does not represent the original GQS classification or a validated categorical threshold.

For mDISCERN, the five-item instrument yields a total score ranging from 0 to 5, with higher scores indicating greater reliability [26]. For the exploratory dichotomization, scores of 1–3 were grouped as lower and scores of 4–5 as higher reliability; this grouping was defined a priori for the present exploratory analysis. For JAMA, the original instrument consists of four binary benchmark criteria scored from 0 to 4 [36] and does not establish a validated categorical cut-off; therefore, scores of 1–2 were classified as lower and scores of 3–4 as higher fulfillment for the present exploratory analysis; this dichotomization was therefore an investigator-defined exploratory grouping. The potential limitations of dichotomizing ordinal variables, including loss of information and reduced statistical power, were considered during interpretation of the findings. A significance level of 5% (*p* < 0.05) was adopted for all analyses.

Because the three instruments were applied using study-specific contextual operational definitions, the statistical analyses evaluate the performance of these contextually adapted applications within the present sample and should not be interpreted as psychometric validation of the original instruments or of the study-specific adaptations.

## Results

### Assessment of inter-rater reliability

The ICC analysis demonstrated excellent inter-rater agreement, with an ICC value of 0.946 (95% CI: 0.913–0.967) for the three evaluated instruments. This high level of agreement indicates that the evaluators applied the assessment criteria consistently across all three evaluation instruments used in the study [15].

Cronbach’s alpha analysis showed high internal consistency between evaluator scores. For the GQS scale, Cronbach’s alpha was 0.913, indicating excellent internal consistency between evaluators.

### GQS scores

The mean score obtained using the contextually adapted GQS application was 4.19 (± 0.78), corresponding to a predominantly high GQS score distribution under the contextually adapted scoring framework. Among the 47 analyzed videos, 20 (42.6%) received the maximum score of 5, 19 (40.4%) received a score of 4, 7 (14.9%) received a score of 3, and 1 (2.1%) received a score of 2. No video received the minimum score of 1, reinforcing the predominance of high-quality materials (Table 1). Overall, 39 videos (83.0%) obtained GQS scores of 4 or 5, indicating good to excellent educational quality.

**Table 1:** Descriptive analysis of the evaluated videos according to the GQS scale criteria (*n* = 47)

Quality Classification	Frequency	Percentage (%)	Valid Percentage (%)	Cumulative Percentage (%)
Very Poor	0	0.0	0.0	0.0
Poor	1	2.1	2.1	2.1
Moderate	7	14.9	14.9	17.0
Good	19	40.4	40.4	57.4
Excellent	20	42.6	42.6	100.0
Total	47	100.0	100.0	—
Mean score: 4.19 (± 0.78)				

*GQS* Global Quality Scale

### JAMA scores

The mean score obtained using the contextually adapted JAMA application was 2.43 ± 1.06. Among the individual JAMA benchmark criteria, 39 videos (83.0%) met the Authorship criterion, 34 (72.3%) fulfilled Attribution, 30 (64.7%) fulfilled Disclosure, and 17 (36.2%) fulfilled Currency (Table 2). Overall, 26 videos (55.3%) met at least three of the four JAMA benchmark criteria.

**Table 2:** Distribution of videos according to the JAMA scale criteria (*n* = 47)

JAMA Criterion	Response	Frequency (%)
Authorship Presence	Yes / No	83.0 / 17.0
Attribution (sources)	Yes / No	72.3 / 27.7
Disclosure (conflict of interest)	Yes / No	64.7 / 35.3
Currency of information	Yes / No	36.2 / 63.8
Final JAMA score (0–4)	Score	
	0	0.0
	1	24.4
	2	21.3
	3	34.0
	4	21.3
Mean score: 2.43 (± 1.06)		

### mDISCERN scores

The mean score obtained using the contextually adapted mDISCERN application was 2.50 ± 0.90. All analyzed videos (47/47; 100%) fulfilled the study-specific contextual criterion corresponding to the mDISCERN item concerning balance/impartiality, whereas 9 videos (19.1%) provided additional sources of information and 1 video (2.1%) addressed areas of uncertainty. The distribution of mDISCERN scores showed that 18 videos (38.3%) received a score of 2, 18 (38.3%) received 3, 3 (6.4%) received 4, and 1 (2.1%) received the maximum score of 5 (Table 3).

**Table 3:** Distribution of videos according to the mDISCERN scale criteria (*n* = 47)

mDISCERN Criterion	Response	Frequency (%)
Objectives Achieved – Criterion (1)	Yes / No	49.9 / 51.1
Reliable Sources – Criterion (2)	Yes / No	72.3 / 27.7
Balanced and Unbiased Information – Criterion (3)	Yes / No	100.0 / 0.0
Sources Listed for Patient Reference – Criterion (4)	Yes / No	19.1 / 80.9
Areas of Uncertainty – Criterion (5)	Yes / No	2.1 / 97.9
Final mDISCERN Score (0–5)	Score	
	1	14.9
	2	38.3
	3	38.3
	4	6.4
	5	2.1
Mean score: 2.50 (± 0.90)		

### Descriptive analysis of differences among author types across evaluation scales

The videos were initially classified into six predefined source categories. After applying the eligibility criteria, four categories were represented in the final sample: Academic Institutions (*n* = 10; 21.3%), Professional Health Organizations (*n* = 5; 10.6%), Healthcare Professionals (*n* = 16; 34.0%), and Other Individual Users (*n* = 16; 34.0%). No eligible videos were identified from Dental Information Sources or Non-Governmental Organizations; therefore, these categories were not included in the comparative analyses because they contained zero observations.

**Fig. 2:**
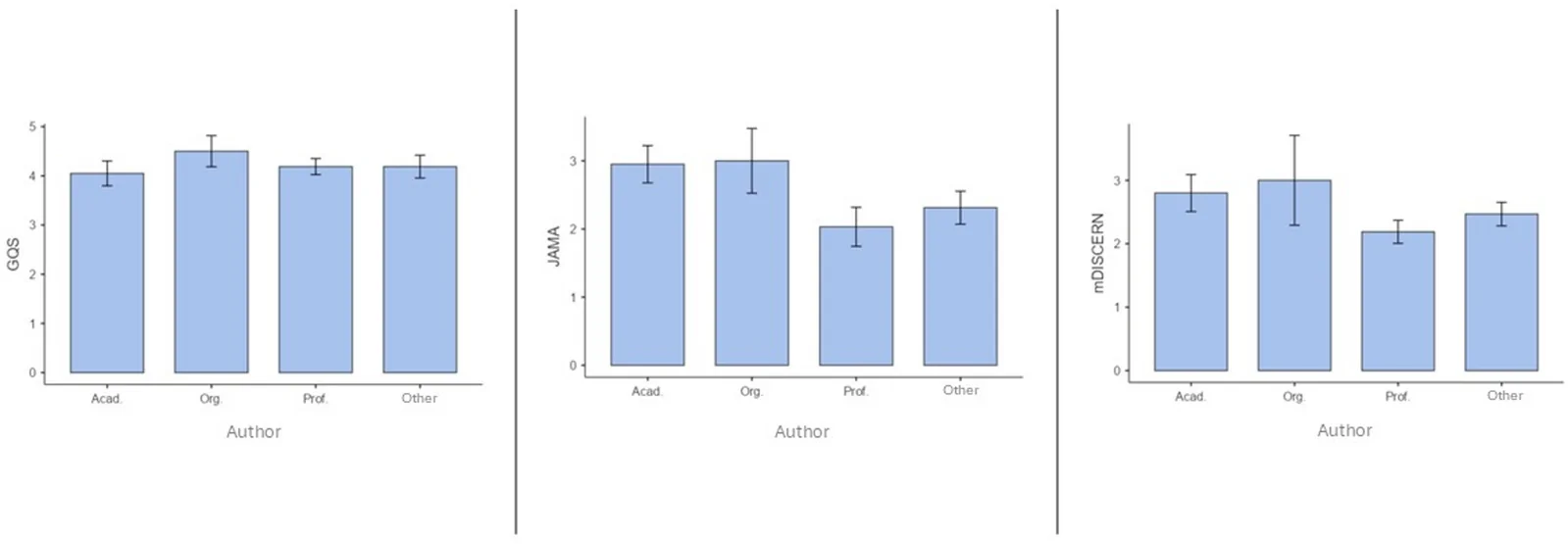
Bar chart showing the mean GQS, JAMA and mDISCERN scores according to the different author groups. The figure highlights variations in content quality among videos produced by academic institutions, professional health organizations, healthcare professionals, and other individual users

The mean scores according to author type are presented in Fig. 2. Videos produced by Academic Institutions achieved mean scores of 4.05 ± 0.80 (GQS), 2.95 ± 0.86 (JAMA), and 2.80 ± 0.92 (mDISCERN). Professional Health Organizations obtained mean scores of 4.50 ± 0.07, 3.00 ± 1.06, and 3.00 ± 1.58, respectively. Healthcare Professionals achieved mean scores of 4.19 ± 0.65, 2.03 ± 1.15, and 2.19 ± 0.73, whereas Other Individual Users obtained mean scores of 4.19 ± 0.92, 2.31 ± 0.96, and 2.47 ± 0.74 for the GQS, JAMA, and mDISCERN scales, respectively.

### Comparison of GQS, JAMA, and mDISCERN scores according to author categories

No statistically significant differences were observed among author categories for any of the evaluated scales. For the GQS, the Kruskal–Wallis test yielded H(3) = 1.271, *p* = 0.736, with a negligible effect size (ε² = 0.028). For the JAMA scale, the test yielded H(3) = 6.079, *p* = 0.108, with a moderate effect size (ε² = 0.132). For the mDISCERN scale, one-way ANOVA yielded F(3, 43) = 1.582, *p* = 0.208, η² = 0.099, ω² = 0.036 (Table 4).

**Table 4:** Comparison of GQS, JAMA and mDISCERN scores among author categories

Scale	Test	Statistic	df	*p*	Effect size
GQS	Kruskal–Wallis	H = 1.271	3	0.736	ε² = 0.028
JAMA	Kruskal–Wallis	H = 6.079	3	0.108	ε² = 0.132
mDISCERN	One-way ANOVA	F = 1.582	3, 43	0.208	η² = 0.099

Assumptions were verified with the Shapiro–Wilk and Levene tests (see Methods)

ε² = epsilon-squared; η² = eta-squared. For the mDISCERN scale, ω² = 0.036

Mean scores and their 95% confidence intervals by author category are presented in Table 5. The intervals were wide, particularly for professional health organizations, which comprised five videos (mDISCERN: 3.00, 95% CI 1.04–4.96). Although none of the comparisons reached statistical significance, the observed effect sizes for the JAMA and mDISCERN scales were of moderate magnitude according to conventional benchmarks; however, the corresponding confidence intervals were wide, indicating substantial uncertainty around these estimates.

**Table 5:** Mean scores by author category, with 95% confidence intervals

Author category	*n*	GQS	JAMA	mDISCERN
Academic institutions	10	4.05 (3.48–4.62)	2.95 (2.33–3.57)	2.80 (2.14–3.46)
Professional health organizations	5	4.50 (3.62–5.38)	3.00 (1.68–4.32)	3.00 (1.04–4.96)
Healthcare professionals	16	4.19 (3.84–4.54)	2.03 (1.42–2.64)	2.19 (1.80–2.58)
Other individual users	16	4.19 (3.69–4.68)	2.31 (1.80–2.83)	2.47 (2.07–2.86)

Values are means with 95% confidence intervals in parentheses

### Correlation between evaluation scales

No statistically significant correlations were identified between the GQS and the JAMA scale (ρ = −0.062, 95% CI − 0.343 to 0.229, *p* = 0.679) or between the GQS and the mDISCERN scale (ρ = 0.087, 95% CI − 0.206 to 0.364, *p* = 0.562). The same pattern was observed when the dichotomized versions of the scales were analysed (*p* > 0.05 in all comparisons). A significant positive correlation of moderate magnitude was observed between the JAMA and mDISCERN scales (ρ = 0.502, 95% CI 0.251 to 0.690, *p* < 0.001) (Table 6).

**Table 6:** Spearman’s correlation coefficients among the GQS, JAMA and mDISCERN scales

Comparison	ρ	95% CI	*p*
GQS × JAMA	−0.062	−0.343 to 0.229	0.679
GQS × mDISCERN	0.087	−0.206 to 0.364	0.562
JAMA × mDISCERN	0.502	0.251 to 0.690	< 0.001

*ρ* Spearman’s rank correlation coefficient, *CI* Confidence interval

## Discussion

The present study evaluated the educational quality, credibility, and reliability of 47 YouTube videos addressing healthcare waste management relevant to dentistry, using contextually adapted applications of the GQS, JAMA benchmark criteria, and mDISCERN. Overall, the findings indicate that the videos retrieved under the specified search strategy during the June 2025 data collection period varied in their levels of educational quality, transparency, and reliability. Although the observed characteristics may have implications for the use of online content in dental education, the present study did not directly evaluate educational effectiveness, knowledge acquisition, learning outcomes, professional competence, or changes in healthcare waste management practices. Therefore, the findings should be interpreted as an assessment of the characteristics of the analyzed online content rather than as evidence that YouTube videos improve learning or professional training. In this context, the potential educational use of such content should be considered an implication that warrants further investigation through studies involving direct assessment of educational outcomes.

The relatively favorable educational quality observed in the evaluated videos may have several possible explanations. One possible explanation is that healthcare waste management involves relatively well-established technical recommendations and standardized procedures. Regulatory documents, particularly ANVISA Resolution RDC No. 222/2018, provide structured guidance regarding classification, segregation, storage, and disposal practices. The availability of such structured guidance could be related to the relatively favorable educational characteristics observed in some videos; however, this possibility was not directly evaluated in the present study, and these findings should not be interpreted as evidence that the videos effectively promote learning or improve professional knowledge or practice. Therefore, the observed GQS scores should not be interpreted as evidence that the regulatory framework directly influenced the quality of the videos. Nevertheless, the widespread availability of standardized information does not necessarily guarantee that online materials provide adequate scientific support, transparency regarding sources, or updated references.

This distinction was evident in the relationship between perceived educational quality and the dimensions assessed by JAMA and mDISCERN. Although many videos appeared clear, organized, and appropriate for learning purposes, these characteristics alone may not necessarily reflect the methodological rigor or the reliability of the information presented. The moderate credibility and reliability observed in the evaluated content indicate that some videos were able to communicate basic concepts effectively while lacking essential characteristics of trustworthy health information, such as explicit authorship, citation of supporting references, disclosure of evidence sources, discussion of uncertainties, and updated supporting evidence. This discrepancy could be related, among other possible explanations, to characteristics of YouTube as an open-access platform. The platform allows users to publish and access content with varying levels of scientific documentation and educational structure. However, the present study did not assess the factors underlying video production, publication, or audience engagement and therefore cannot determine whether the open nature of the platform contributed to the observed differences between educational presentation and credibility/reliability.

This interpretation is supported by the low performance observed in specific transparency-related criteria. Only 36.2% of the videos fulfilled the study-specific Currency criterion, which required explicit reference to Resolution RDC No. 222/2018. Only 19.1% of the videos fulfilled the study-specific mDISCERN criterion requiring explicit citation or identification of information sources, and only 2.1% discussed areas of uncertainty related to the topic. Together, these findings indicate that, within the videos retrieved under the present search strategy, several materials provided limited evidence of scientific support according to the criteria applied in this study. The accessibility of YouTube may make such content available to a broad audience; however, whether this accessibility facilitates educational use or influences learners was not assessed in the present study. Similar limitations have been reported in previous evaluations of health-related content available on YouTube, demonstrating that visually appealing and accessible materials may still present important deficiencies regarding scientific transparency and evidence support [25, 27–31 ].

The limited availability of references and updated information represents an important concern in the context of healthcare education. Unlike traditional academic resources, YouTube content is not routinely subjected to peer review or formal educational validation before publication. Consequently, individuals who use such videos for educational purposes could potentially encounter materials that are relatively easy to understand but insufficiently supported by scientific evidence. However, the present study did not evaluate how learners select, interpret, or use these materials, nor whether exposure to them affects learning or professional decision-making. This issue is particularly relevant for healthcare waste management, in which inadequate information may influence professional practices related to biosafety, environmental protection, and regulatory compliance. Therefore, although YouTube may facilitate access to educational materials, its use should be accompanied by strategies that promote information verification, critical appraisal, and evidence-based decision-making among students and healthcare professionals [3, 29, 31].

These results are comparable to those reported by Azevedo et al. (2024) [11], who found a mean mDISCERN score of 2.3 for the analyzed videos, with only 10% of them providing references to viewers. Likewise, previous studies have consistently shown that health-related educational content available on YouTube frequently lacks adequate scientific support despite being visually attractive and well organized [28, 29]. Taken together, these findings reinforce that educational quality, credibility, and reliability represent complementary -but distinct- dimensions of digital educational resources. Consequently, the evaluation of YouTube videos intended for educational purposes should not rely exclusively on presentation quality or perceived usefulness but should also consider transparency, evidence support, and methodological reliability, particularly in technical fields such as healthcare waste management.

No statistically significant differences were detected among the represented author categories for GQS, JAMA, or mDISCERN scores in the present sample. These findings should not, however, be interpreted as evidence that the source of publication is equivalent across categories or that authorship does not influence the quality, credibility, or reliability of online content. The relatively small number of videos in some categories, particularly Professional Health Organizations (*n* = 5), resulted in wide confidence intervals and limited precision of the corresponding estimates. Although the JAMA and mDISCERN analyses showed effect sizes of dence intervals indicate substantial imprecision in the estimated differences among source categories. Therefore, the non-significant findings should not be interpreted as evidence that differences or associations are absent. Rather, they indicate that the present data provide insufficiently precise evidence to determine the presence or magnitude of systematic differences among the represented source categories. These findings should consequently be interpreted with caution, considering the uncertainty associated with subgroup size and estimate precision [1, 2].

However, the absence of statistically significant differences should not be interpreted as evidence that authorship is irrelevant or that different source categories perform similarly. On digital platforms such as YouTube, institutional affiliation may influence users’ perceptions of credibility, but the present study was not designed to establish whether source type systematically determines educational quality, credibility, or reliability. The relatively small subgroup sizes and the resulting uncertainty in the estimates limit the precision with which differences among source categories can be assessed. Accordingly, objective assessment criteria remain important for identifying online educational resources, while the potential influence of authorship or institutional affiliation should be investigated in larger samples with adequate representation of different source categories [25, 28, 29, 31].

The absence of statistically significant differences in the present sample does not provide sufficient evidence to establish whether institutional affiliation or professional source type is associated with higher reliability. Although variation in scores was observed among author groups, the available sample does not allow definitive conclusions regarding systematic differences between source categories. The wide confidence intervals observed for some groups, particularly those with few videos, indicate substantial uncertainty around the estimated group means. Therefore, the present findings should be interpreted as insufficient evidence to determine whether authorship alone is associated with differences in educational quality, credibility, or reliability, and larger studies with more balanced representation across source categories are needed to investigate this question [15, 28–31].

Beyond the characteristics of content producers, the assessment of digital educational resources also requires consideration of multiple quality dimensions. The correlation pattern observed among the evaluation instruments indicates that the different dimensions assessed in the present study should be interpreted as complementary but distinct constructs. The positive association between the contextually adapted JAMA and mDISCERN scores indicates that higher scores on one assessment approach were associated with higher scores on the other in the present sample. One possible explanation is that both approaches include criteria related to the documentation and reliability of health information, such as authorship, information sources, and references. However, this interpretation is hypothetical because the present study did not directly evaluate the mechanisms underlying this association or determine which specific criteria contributed to the observed correlation.

Therefore, the observed association should not be interpreted as evidence that transparency itself leads to greater reliability or trustworthiness. Because both instruments were applied using study-specific contextual operational definitions, this association may also partly reflect the measurement approach adopted in the present study. Thus, the observed correlation should be interpreted as an association between the scores obtained under the present operational framework, rather than as definitive evidence of an intrinsic relationship between credibility and reliability in the analyzed videos.

In contrast, no statistically significant associations were detected between GQS scores and the JAMA or mDISCERN scores in the present sample. These findings indicate that a statistically significant relationship was not detected under the conditions of the study, but they should not be interpreted as evidence that no relationship exists between educational presentation and measures of credibility or reliability. The observed correlations were weak, and their confidence intervals included values compatible with both weak negative and weak positive associations. Moreover, the contextual operationalization of the GQS may have influenced the distribution and variability of GQS scores and, consequently, the observed correlations with the other instruments. Therefore, the absence of statistical significance should be interpreted as uncertainty regarding the presence or magnitude of an association rather than as evidence of the absence of a relationship. Accordingly, educational presentation and perceived usefulness should not be assumed to correspond to scientific accuracy or informational reliability based solely on the present findings [25].

Similar findings were reported by Naik et al. (2025) [15], who observed a positive correlation between JAMA and DISCERN, whereas GQS showed weak associations with credibility-related measures. Taken together, these findings suggest that the relationship between perceived educational quality and credibility-related dimensions may be complex; however, the absence of statistically significant associations in the present study should not be interpreted as evidence that no relationship exists. Larger samples and studies specifically designed to estimate the magnitude and precision of these associations are warranted [31].

The categorized analyses performed in the present study were conducted exclusively as exploratory approaches to facilitate the interpretation of potential relationships among the assessment instruments. Although categorization into high-and-low quality groups may improve clinical interpretability, dichotomization of ordinal variables may reduce statistical power and result in loss of information contained within the original scoring systems. Therefore, continuous scores were considered the primary representation of the evaluated constructs, and findings derived from categorized variables should be interpreted with caution.

An important methodological consideration concerns the contextual application of the GQS, JAMA benchmark criteria, and mDISCERN. Although the original names, general structures, and scoring ranges of the instruments were retained, selected criteria were operationalized using predefined rules developed specifically for the Brazilian healthcare waste management context. These contextual operational definitions were intended to improve reproducibility and reduce subjectivity during evaluator judgment; however, they may also have influenced the constructs represented by the resulting scores. In particular, the JAMA Currency criterion was operationalized according to explicit reference to Resolution RDC No. 222/2018, while selected mDISCERN criteria were interpreted according to predefined contextual rules involving regulatory alignment and the identification of uncertainty or complementary information. Similarly, contextual healthcare waste management indicators were used to support the interpretation of educational relevance in the GQS assessment. Therefore, the instruments should be understood in the present study as contextually adapted applications rather than unmodified applications of their original validated definitions. Because these operational adaptations have not undergone independent psychometric validation, their construct validity, measurement equivalence, and direct comparability with studies using the original instruments remain uncertain. The excellent inter-rater agreement observed in the present study indicates consistency between evaluators but should not be interpreted as evidence of validity of the contextual adaptations. Future studies should investigate the psychometric properties of these operational definitions or develop and validate a specific instrument for assessing digital educational content on healthcare waste management relevant to dentistry.

Additionally, the relatively high GQS scores observed in the sample could be consistent with a possible ceiling effect, potentially related to the contextual adaptation of the scoring approach. Because the adapted criteria emphasized the presence of predefined healthcare waste management topics, videos addressing these domains may have received higher scores even when other educational characteristics were less robust. This is a possible explanation rather than a mechanism demonstrated by the present study, because the study was not designed to formally test the causes of the observed score distribution. Furthermore, although the calibration procedures and contextual operational definitions may have improved consistency among evaluators, they may also have influenced the measurement of the aspects assessed by the GQS and contributed to the relatively restricted variability observed in scores. Because these operational definitions have not been independently validated, the possibility of a measurement-related ceiling effect cannot be excluded.

These considerations should be taken into account when interpreting the relationship between GQS scores and the other indicators of information quality assessed in the present study. More broadly, the contextual operationalization of the GQS, JAMA benchmark criteria, and mDISCERN may have influenced not only the interpretation of individual scores, but also the mean scores, score distributions, comparisons among author categories, and correlations between instruments. Consequently, the statistical relationships observed in the present study may partly reflect the measurement approach adopted by the investigators and the specific operational definitions used, rather than solely intrinsic characteristics of the analyzed videos. This consideration is particularly relevant when interpreting the observed scores as indicators of educational quality, credibility, or reliability. Because the contextual adaptations have not undergone independent psychometric validation, these findings should be interpreted as measurements obtained within the specific methodological framework of the present study rather than as definitive, instrument-independent measures of these characteristics.

In addition to the methodological considerations related to the assessment instruments, some aspects of the study design should also be acknowledged. The decision to analyze only videos produced in Brazilian Portuguese may represent a limitation of the present study, as it restricts comparisons with content produced in other languages and countries, which may limit the generalizability of the findings to the global context of online health education [11, 15]. The absence of statistically significant differences should not necessarily be interpreted as evidence that no differences exist, particularly considering the moderate effect sizes observed for the JAMA and mDISCERN analyses. The findings should therefore be interpreted within the context of Brazilian Portuguese YouTube content and may not be directly generalizable to other languages, countries, or digital platforms.

Selection bias represents another potential limitation, as only the first 60 videos retrieved according to YouTube’s relevance-based search ordering for each search term were included in the initial screening process [7, 21, 26]. Although this strategy aimed to evaluate videos with greater visibility within the platform’s search results, it may not represent the full diversity of healthcare waste management videos available on YouTube, particularly content with lower ranking positions but potentially different educational characteristics.

Furthermore, although strategies such as the use of an incognito browser mode, cleared cache and cookies, and a newly created YouTube account were adopted to minimize personalization effects, algorithmic bias cannot be completely excluded. YouTube recommendations and search rankings may still vary according to geographic location, language settings, user behavior, and temporal changes in platform’s ranking algorithms. Therefore, the findings should be interpreted as a representation of available content during the data collection period rather than a permanent characterization of the platform.

Another limitation was the absence of engagement-related variables, such as views, likes, dislikes, comments, and user interaction patterns. These indicators could provide additional information regarding audience perception and the relationship between popularity and educational quality [32]. Likewise, users’ perceptions regarding the usefulness of the videos, an essential factor in the assessment of educational materials, were not considered in the present study, limiting the understanding of the practical impact of these contents on the target audience [29]. Based on these limitations, future studies should consider larger sample sizes, incorporate engagement-related variables, include user perceptions, and evaluate additional digital platforms beyond YouTube, thereby expanding the scope and robustness of the analyses [31].

An additional important limitation is that the present study evaluated the characteristics of online videos but did not directly assess their educational effectiveness. No measures of knowledge acquisition, learning outcomes, retention of information, professional competence, behavioral change, or changes in healthcare waste management practices were collected. Therefore, the observed GQS, JAMA, and mDISCERN scores cannot be interpreted as evidence that the analyzed videos improve learning or professional performance. Future studies should directly assess educational outcomes among students or healthcare professionals exposed to such content to determine whether the characteristics identified in the present study translate into measurable educational benefits.

Furthermore, the interpretation of findings from YouTube-based studies should consider the dynamic nature of the platform itself. Content availability, user engagement, and search rankings may change over time, meaning that the characteristics of available videos can vary according to the period of data collection. Therefore, the findings of this study should be viewed as a representation of YouTube content during the data collection period (June 2025). Subsequent investigations using repeated assessments over time may help determine the stability of educational content quality and evaluate how changes in platform dynamics influence the availability and visibility of digital educational resources.

Despite these limitations, the present findings may have implications for dental education and the development of online health-related resources. Considering the increasing incorporation of digital media into educational environments, academic institutions and professional organizations could contribute to the availability of evidence-based audiovisual materials by developing and curating content addressing healthcare waste management. Such initiatives may improve the accessibility and transparency of online information; however, whether exposure to such content results in improved knowledge, learning, professional competence, or safer healthcare waste management practices was not evaluated in the present study and remains an empirical question for future research [2, 3, 33, 34].

In this context, the findings indicate specific gaps in the analyzed videos, particularly regarding the currency of information, citation of supporting sources, and discussion of uncertainty. These findings may inform the development and curation of future audiovisual materials by highlighting aspects that could be addressed to improve the transparency and informational support of online content [28]. The dissemination of updated, evidence-based, and accessible audiovisual materials may therefore represent a promising approach for providing more transparent online information; however, its effectiveness in strengthening digital health education or improving learners’ knowledge and competence remains to be established through direct educational evaluation. Higher education institutions and professional councils may have a relevant role in developing and curating such resources while also promoting critical appraisal skills among healthcare students and professionals [33].

Overall, the present study identified characteristics of YouTube videos that may be relevant to the appraisal of online information concerning healthcare waste management in dentistry. The analyzed content demonstrated satisfactory educational quality but only moderate credibility and reliability, indicating that clarity and perceived educational usefulness as assessed by the GQS should not be considered sufficient indicators of scientific support. The observed characteristics indicate that some of the analyzed videos could potentially serve as supplementary informational resources; however, whether they function effectively as educational resources cannot be determined from the present findings. The present study did not evaluate whether exposure to these videos improves learning, knowledge acquisition, professional competence, or healthcare waste management practices. Therefore, the educational role of YouTube content in this context remains an empirical question rather than an effect demonstrated by the present study. Future studies involving students and healthcare professionals, with direct measures of knowledge, learning outcomes, competence, or behavioral change, are needed to determine the actual educational effectiveness of such content [25, 33–36].

## Conclusion

Most of the 47 YouTube videos evaluated in this study exhibited satisfactory educational quality, although only moderate levels of credibility and reliability were observed. No statistically significant differences in scores were identified across the represented content-source categories for any of the assessment instruments. Nevertheless, the small subgroup sizes constrain the precision and interpretability of these comparisons. Correlation analysis identified a positive association between the scores derived from the contextually adapted JAMA and mDISCERN applications, whereas the contextually adapted GQS scores were not significantly associated with either instrument. Importantly, the absence of statistically significant differences or associations in this sample should not be interpreted as evidence of equivalence among source categories or as demonstrating that no relationship exists between GQS and the other assessment instruments.

Taken together, these findings highlight the value of critically appraising online health-related content through complementary approaches that capture distinct dimensions of quality, particularly in open and heterogeneous digital environments such as YouTube. The results reflect the characteristics of videos retrieved according to the predefined search strategy during the June 2025 data collection period and, therefore, cannot be generalized to YouTube content as a whole. Nor should they be construed as evidence of educational effectiveness, learning outcomes, professional competence, or changes in healthcare waste management practices.

The interpretation of these findings also requires consideration of an important methodological constraint. The GQS, JAMA benchmark criteria, and mDISCERN were applied according to study-specific contextual operational definitions that have not undergone independent psychometric validation. These adaptations may have affected score distributions, comparisons across source categories, and correlations between instruments. Consequently, the statistical patterns observed may reflect, at least in part, the measurement framework adopted in this study rather than solely the intrinsic characteristics of the videos evaluated. Comparisons with studies employing the original instruments under their unmodified validated definitions should therefore be undertaken cautiously.

Ultimately, whether the characteristics identified in these videos translate into meaningful and measurable educational benefits remains unresolved. Future investigations should move beyond assessments of content quality alone by incorporating direct measures of educational outcomes, while using larger samples with adequate representation of the relevant content-source categories. Such approaches may provide stronger evidence regarding the educational value of health-related YouTube content and its potential implications for healthcare waste management. 

## Supplementary Information

Supplementary Material 1.

Supplementary Material 2.

Supplementary Material 3.

## Data Availability

All data generated or analyzed during this study are included in this published article [and its supplementary information files].
