# Supplementary material for: Educational quality of YouTube content on healthcare waste management relevant to dentistry: a cross-sectional study
Source: BMC Oral Health. 2026 Oct 12;26:1995. doi: 10.1186/s12903-026-10137-4 (PMC13662963; doi:10.1186/s12903-026-10137-4)
Supplement: Supplementary file 1 [file 12903_2026_10137_MOESM1_ESM.docx]

Supplementary material 1. Evaluation Criteria of the GQS Scale.

| Score | Criterion |
| --- | --- |
| 1 | Poor quality, poor flow of the website/video, most information missing, not useful for the target audience. |
| 2 | Generally poor quality and poor flow, some information listed but many important topics missing, of very limited use to the target audience. |
| 3 | Moderate quality, suboptimal flow, some important information adequately discussed while others are poorly addressed, of limited usefulness to the target audience. |
| 4 | Good quality and generally good flow, most relevant information listed, although some topics are not covered, useful for the target audience. |
| 5 | Excellent quality and excellent flow, highly useful for the target audience. |
