# Supplementary material for: Educational quality of YouTube content on healthcare waste management relevant to dentistry: a cross-sectional study
Source: BMC Oral Health. 2026 Oct 12;26:1995. doi: 10.1186/s12903-026-10137-4 (PMC13662963; doi:10.1186/s12903-026-10137-4)
Supplement: Supplementary file 2 [file 12903_2026_10137_MOESM2_ESM.docx]

Supplementary material 2. Evaluation Criteria of the JAMA Scale.

| Criterion | Yes or No |
| --- | --- |
| Authorship – Presence of the names of authors and contributors, including their respective affiliations and credentials, ensuring author credibility. |  |
| Attribution – Presence of references and sources for all described content, confirming proper citation of sources and relevant copyright information. |  |
| Disclosure – Disclosure of ownership, sponsors, advertising policies, and commercial conflicts of interest. |  |
| Currency – Assessment of the timeliness of the information, including the dates when the content was posted and updated. |  |
