# Supplementary material for: Educational quality of YouTube content on healthcare waste management relevant to dentistry: a cross-sectional study
Source: BMC Oral Health. 2026 Oct 12;26:1995. doi: 10.1186/s12903-026-10137-4 (PMC13662963; doi:10.1186/s12903-026-10137-4)
Supplement: Supplementary file 3 [file 12903_2026_10137_MOESM3_ESM.docx]

Supplementary material 3. Evaluation Criteria of the mDISCERN Scale.

| Criterion | Yes or No |
| --- | --- |
| (1) Are the objectives clear and achieved? |  |
| (2) Are reliable sources of information used? (e.g., cited publications, speaker is a dentistry expert?) |  |
| (3) Is the presented information balanced and unbiased? |  |
| (4) Are additional sources of information listed for patient reference? |  |
| (5) Are areas of uncertainty regarding the topic mentioned? |  |
